# Oral anticoagulant underutilization among elderly patients with atrial fibrillation: insights from the United States Medicare database

**DOI:** 10.1007/s10840-022-01274-1

**Published:** 2022-07-09

**Authors:** Muhammad Bilal Munir, Patrick Hlavacek, Allison Keshishian, Jennifer D. Guo, Rajesh Mallampati, Mauricio Ferri, Cristina Russ, Birol Emir, Matthew Cato, Huseyin Yuce, Jonathan C. Hsu

**Affiliations:** 1 Section of Electrophysiology, Division of Cardiology, University of California San Diego School of Medicine, La Jolla, CA USA; 2 Section of Electrophysiology, Division of Cardiology, University of California Davis School of Medicine, Sacramento, CA USA; 3 Pfizer, New York, NY USA; 4 STATinMED Research, Ann Arbor, MI USA; 5 Bristol Myers Squibb, Lawrenceville, NJ USA; 6 New York City College of Technology, City University of New York, New York, NY USA

**Keywords:** Oral anticoagulant therapy, Elderly, Atrial Fibrillation, Direct oral anticoagulant, Underutilization

## Abstract

**Background:**

Oral anticoagulants (OACs) mitigate stroke risk in patients with atrial fibrillation (AF). The study aim was to analyze prevalence and predictors of OAC underutilization.

**Methods:**

Newly diagnosed AF patients with a CHA_2_DS_2_-VASc score ≥ 2 were identified from the US CMS Database (January 1, 2013–December 31, 2017). Patients were stratified based on having an OAC prescription versus not and the OAC prescription group was stratified by direct OAC (DOACs) versus warfarin. Multivariable logistic regression models were used to examine predictors of OAC underutilization.

**Results:**

Among 1,204,507 identified AF patients, 617,611 patients (51.3%) were not prescribed an OAC during follow-up (mean: 2.4 years), and 586,896 patients (48.7%) were prescribed an OAC during this period (DOAC: 388,629 [66.2%]; warfarin: 198,267 [33.8%]). Age ≥ 85 years (odds ratio [OR] 0.55, 95% confidence interval [CI] 0.55–0.56), female sex (OR 0.96, 95% CI 0.95–0.96), Black race (OR 0.78, 95% CI 0.77–0.79) and comorbidities such as gastrointestinal (GI; OR 0.43, 95% CI 0.41–0.44) and intracranial bleeding (OR 0.29, 95% CI 0.28–0.31) were associated with lower utilization of OACs. Furthermore, age ≥ 85 years (OR 0.92, 95% CI 0.91–0.94), Black race (OR 0.78, 95% CI 0.76–0.80), ischemic stroke (OR 0.77, 95% CI 0.75–0.80), GI bleeding (OR 0.73, 95% CI 0.68–0.77), and intracranial bleeding (OR 0.72, 95% CI 0.65–0.80) predicted lower use of DOACs versus warfarin.

**Conclusions:**

Although OAC therapy prescription is the standard of care for stroke prevention in AF patients, its overall utilization is still low among Medicare patients ≥ 65 years old, with specific patient characteristics that predict underutilization.

**Supplementary Information:**

The online version contains supplementary material available at 10.1007/s10840-022-01274-1.

## Introduction

Atrial fibrillation (AF) is the most common sustained cardiac arrhythmia encountered in clinical practice. The incidence of AF in the United States (US) is expected to double from 1.2 million in 2010 to 2.6 million in 2030 largely due to an aging population [1–3]. AF is associated with a fivefold increased risk of stroke, and AF-related strokes are associated with worse morbidity and mortality when compared to strokes not related to AF [4, 5].

Oral anticoagulants (OACs) are the standard of care for mitigating stroke risks in AF patients. Due to better safety and efficacy, direct-acting oral anticoagulants (DOACs) are now recommended as first-line treatment compared to warfarin for reduction of stroke in AF patients based on eligible CHA_2_DS_2_-VASc score [1, 6, 7]. Patients ≥ 65with AF are especially prone to ischemic stroke and studies have shown absolute reduction in the risk of stroke in such patients when prescribed OAC therapy [8]. Several earlier studies have shown underutilization of OAC therapy in eligible AF patients. However, most of these studies were done in the era when DOACs were still investigational or assimilating into clinical practice [9–14]. Therefore, we conducted a real-world observational study from a large sample of Medicare patients in order to assess the prevalence and predictors of OAC therapy underutilization among AF patients ≥ 65 years of age at risk of stroke in contemporary practice. We also assessed the trends of warfarin and DOAC utilization over our study time period and the predictors of DOAC therapy prescription (i.e., either apixaban, dabigatran, edoxaban. or rivaroxaban) versus warfarin.

## Methods

### Data source

This was a retrospective cohort study using the United States Centers for Medicare & Medicaid Services (CMS) fee-for-service Medicare dataset (100%) from January 1, 2012 to December 31, 2017. Fee-for-service Medicare is a federal health insurance program that covers over 38 million patients, including those aged ≥ 65 years and other special groups of patients in the US. The database contains medical and pharmacy claims from Medicare data, including inpatient, outpatient, carrier, Part D, skilled nursing facility, home health agency, and durable medical equipment claims. Pharmacy claims are recorded based on the drug dispensed using the National Drug Code coding system.

### Patient selection

Patients were required to be 65 years or older and have ≥ 1 inpatient or ≥ 2 outpatient medical claims (separated by ≥ 7 days) for AF in any diagnosis position. The first AF diagnosis date was designated as the index date for the purposes of our analysis. Patients were required to have a CHA_2_DS_2_-VASc score of ≥ 2 during the 12-month pre-index period (baseline period). This was based on previous AF consensus guidelines which, at the time of practice, recommended OAC therapy prescriptions based on this stroke risk scoring system [1]. In addition, patients were also required to have continuous health plan enrollment with medical and pharmacy benefits during the baseline period and ≥ 6 months after the index date (follow-up period). In order to select only patients with incident AF during the study period, patients with an AF diagnosis prior to the index date were excluded. Patients with medical claims indicating diagnosis of rheumatic mitral valvular heart disease and valve replacement procedure were excluded. In addition, those with pharmacy claims for an OAC therapy prescription (i.e., apixaban, dabigatran, edoxaban, rivaroxaban, or warfarin) during the baseline period were excluded to ensure new OAC use. All relevant International Classification of Disease, 9th/10th revision, clinical modification [ICD-9/10-CM] diagnosis, and procedure codes are presented in Supplemental Table 1.

**Fig. 1:**
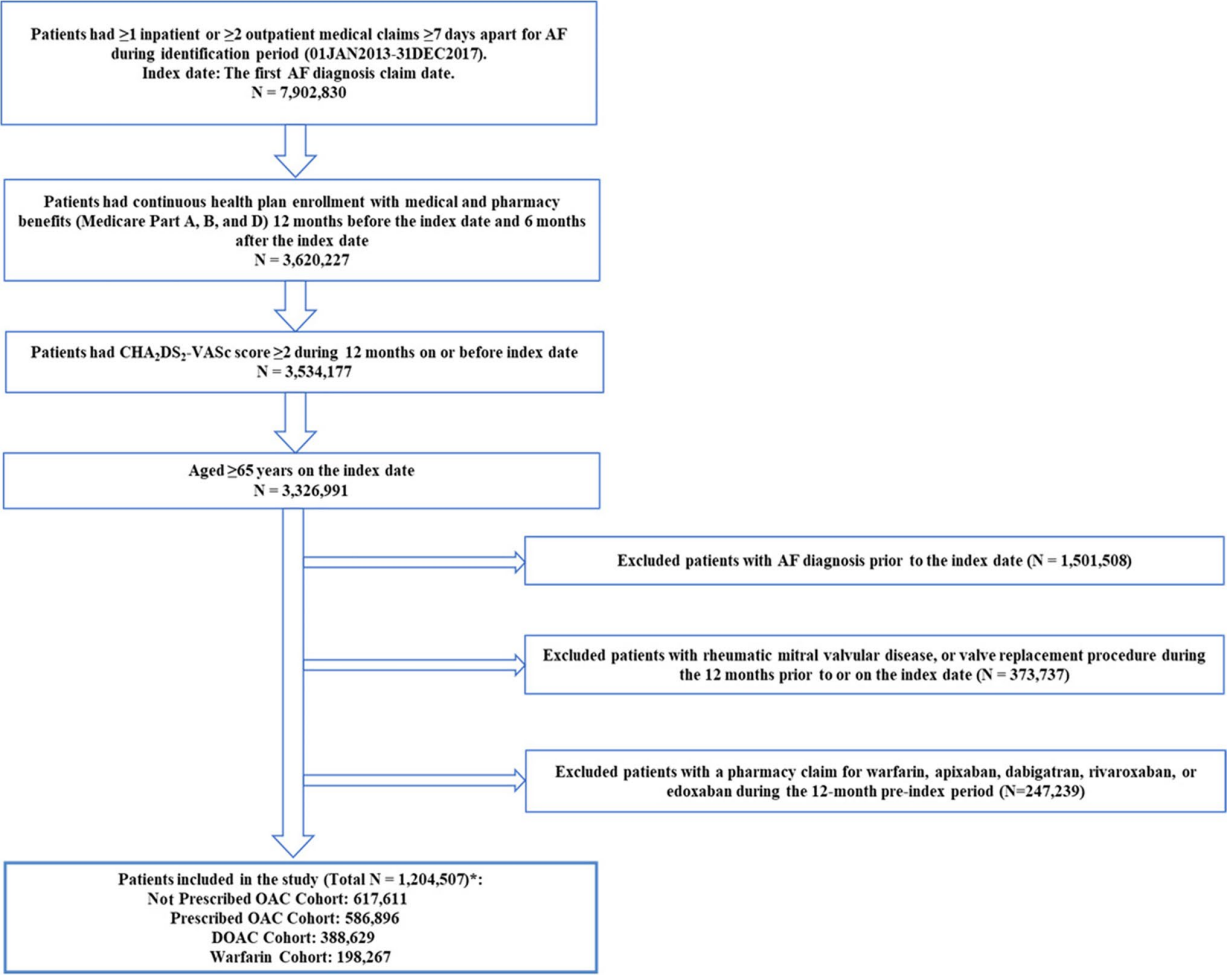
Patient selection criteria. AF, atrial fibrillation; DOAC, direct oral anticoagulants; ICD-9/10-CM: International Classification of Disease 9^th^/10.^th^ Revision Clinical Modification

Patients were assigned to the OAC-prescribed or not OAC-prescribed cohorts based on whether they were ever prescribed OAC therapy at any time on or after the index AF diagnosis (follow-up period). Based on the type of index prescription, patients were assigned to either the DOAC (apixaban, dabigatran, edoxaban, or rivaroxaban) or warfarin sub-cohorts. Figure 1 further depicts detailed patient selection criteria.

### Baseline variables

Patient demographics, clinical characteristics (i.e., clinical risk scores, comorbidities, and medications) and health care utilization (emergency room [ER], office visits, and inpatient admissions) were measured during the 12-month baseline period. Age, sex, and race were measured on the index date. While race is acknowledged here as a social construct, this study assessed race categories based on CMS codes [15]. Patient CHA_2_DS_2_-VASc scores were tabulated to assess stroke risk, while modified, code-based HAS-BLED scores (international normalized ratio [INR], lab values, and self-reported alcohol consumption were not available) were tabulated to evaluate risk of bleeding. Evidence of bleeding and stroke was evaluated during the 12-month baseline period. A history of major bleeding was classified as intracranial (ICH), major gastrointestinal (GI), or other major bleeding based on claims in the inpatient setting in the primary diagnosis position. In addition, non-major bleeding in any clinical setting was evaluated. A history of stroke/systemic embolism (SE) was classified as ischemic stroke and SE based on a primary diagnosis in the inpatient setting. Hemorrhagic stroke was not separately assessed, as it was a component of intracranial bleeding.

### Statistical analysis

All variables were stratified by cohort and analyzed descriptively. Means and standard deviations were provided for continuous variables. Frequencies and percentages were provided for categorical variables. *T*-tests and chi-square tests were conducted to compare cohorts.

Multivariable logistic regression models were constructed to assess the adjusted association of baseline demographics, clinical characteristics, medications, and health care utilization with OAC therapy prescription. All baseline variables were included in the logistic regression model to assess for potential independent predictors, and to adjust for potential confounding (See Table 1 for complete list). Age and sex were forced into the model. Backward stepwise model selection was used with entry and stay thresholds of *p* < 0.15 each to select variables. In the main analysis, age was considered as a categorical variable (65–74, 75–84, ≥ 85 years); however, in a supplemental analysis, age was included in the model as a continuous variable. The presence of interactions between selected study variables of clinical significance were evaluated based on a priori specification. A *P* value < 0.05 was considered significant for main effects, while a *P* value < 0.10 was considered statistically significant for interaction terms. All analyses were conducted using SAS 9.4 [Cary, NC]. Missing data, if any, were not imputed.

**Table 1:** Baseline characteristics of Medicare patients with atrial fibrillation: prescribed versus not prescribed oral anticoagulant therapy

				Prescribed with OAC cohort		
	Prescribed with OAC cohort	Not prescribed with OAC cohort		DOAC cohort	Warfarin cohort	
	*N* = 586,896	*N* = 617,611		*N* = 388,629	*N* = 198,267	
	N/Mean (%/SD)	N/Mean (%/SD)	*p*-value	*N*/mean (%/SD)	*N*/mean (%/SD)	*p*-value
Age^a^	77.9 (7.3)	80.4 (8.7)	< .0001	77.8 (7.2)	78.2 (7.4)	< .0001
65–74 years	212,736 (36.2%)	184,992 (30.0%)	< .0001	143,916 (37.0%)	68,820 (34.7%)	< .0001
75–84 years	252,919 (43.1%)	219,470 (35.5%)	< .0001	167,004 (43.0%)	85,915 (43.3%)	.0084
≥ 85 years	121,241 (20.7%)	213,149 (34.5%)	< .0001	77,709 (20.0%)	43,532 (22.0%)	< .0001
Sex^a^						
Male	263,182 (44.8%)	258,047 (41.8%)	< .0001	174,655 (44.9%)	88,527 (44.7%)	.0340
Female	323,714 (55.2%)	359,564 (58.2%)	< .0001	213,974 (55.1%)	109,740 (55.3%)	.0340
Race^a^						
White	526,355 (89.7%)	532,722 (86.3%)	< .0001	350,403 (90.2%)	175,952 (88.7%)	< .0001
Black	31,823 (5.4%)	47,273 (7.7%)	< .0001	18,572 (4.8%)	13,251 (6.7%)	< .0001
Other	28,718 (4.9%)	37,616 (6.1%)	< .0001	19,654 (5.1%)	9,064 (4.6%)	< .0001
US geographic region^a^						
Northeast	117,328 (20.0%)	117,899 (19.1%)	< .0001	76,436 (19.7%)	40,892 (20.6%)	< .0001
Midwest	156,387 (26.6%)	148,821 (24.1%)	< .0001	91,920 (23.7%)	64,467 (32.5%)	< .0001
South	216,612 (36.9%)	239,362 (38.8%)	< .0001	157,130 (40.4%)	59,482 (30.0%)	< .0001
West	95,617 (16.3%)	110,357 (17.9%)	< .0001	62,579 (16.1%)	33,038 (16.7%)	< .0001
Other	952 (0.2%)	1,172 (0.2%)	.0003	564 (0.1%)	388 (0.2%)	< .0001
Medicaid dual eligibility^a^	142,530 (24.3%)	213,457 (34.6%)	< .0001	86,619 (22.3%)	55,911 (28.2%)	< .0001
Part-D low-income subsidy	158,342 (27.0%)	230,173 (37.3%)	< .0001	96,778 (24.9%)	61,564 (31.1%)	< .0001
Charlson comorbidity index score^a^	2.9 (2.6)	3.4 (2.8)	< .0001	2.8 (2.5)	3.2 (2.7)	< .0001
CHA_2_DS_2_-VASc score	4.5 (1.6)	4.8 (1.6)	< .0001	4.4 (1.6)	4.7 (1.6)	< .0001
2–3	165,347 (28.2%)	143,759 (23.3%)	< .0001	117,395 (30.2%)	47,952 (24.2%)	< .0001
4–5	269,105 (45.9%)	280,725 (45.5%)	< .0001	177,882 (45.8%)	91,223 (46.0%)	.0829
≥ 6	152,444 (26.0%)	193,127 (31.3%)	< .0001	93,352 (24.0%)	59,092 (29.8%)	< .0001
HAS-BLED score^b^	3.3 (1.2)	3.5 (1.3)	< .0001	3.2 (1.2)	3.4 (1.2)	< .0001
0–2	173,236 (29.5%)	150,703 (24.4%)	< .0001	119,908 (30.9%)	53,328 (26.9%)	< .0001
3–4	320,318 (54.6%)	336,331 (54.5%)	.1805	212,710 (54.7%)	107,608 (54.3%)	.0008
≥ 5	93,342 (15.9%)	130,577 (21.1%)	< .0001	56,011 (14.4%)	37,331 (18.8%)	< .0001
Major bleeding^a^						
Gastrointestinal bleeding	4214 (0.7%)	12,640 (2.0%)	< .0001	2282 (0.6%)	1932 (1.0%)	< .0001
Intracranial bleeding	1530 (0.3%)	6108 (1.0%)	< .0001	862 (0.2%)	668 (0.3%)	< .0001
Other major bleeding	4343 (0.7%)	12,446 (2.0%)	< .0001	2226 (0.6%)	2117 (1.1%)	< .0001
Non-major bleeding ^a^	116,788 (19.9%)	151,153 (24.5%)	< .0001	71,445 (18.4%)	45,343 (22.9%)	< .0001
Stroke/systemic embolism ^a^						
Ischemic stroke	22,139 (3.8%)	13,605 (2.2%)	< .0001	12,992 (3.3%)	9147 (4.6%)	< .0001
Systemic embolism	1047 (0.2%)	315 (0.1%)	< .0001	404 (0.1%)	643 (0.3%)	< .0001
Baseline comorbidities^a^						
Obesity	127,003 (21.6%)	99,037 (16.0%)	< .0001	82,922 (21.3%)	44,081 (22.2%)	< .0001
Congestive heart failure	156,480 (26.7%)	189,105 (30.6%)	< .0001	94,513 (24.3%)	61,967 (31.3%)	< .0001
Diabetes	226,134 (38.5%)	244,240 (39.5%)	< .0001	144,033 (37.1%)	82,101 (41.4%)	< .0001
Hypertension	523,486 (89.2%)	544,917 (88.2%)	< .0001	346,085 (89.1%)	177,401 (89.5%)	< .0001
Chronic obstructive pulmonary disease	139,003 (23.7%)	178,607 (28.9%)	< .0001	87,941 (22.6%)	51,062 (25.8%)	< .0001
Renal disease	138,975 (23.7%)	179,699 (29.1%)	< .0001	82,353 (21.2%)	56,622 (28.6%)	< .0001
Myocardial infarction	77,994 (13.3%)	98,318 (15.9%)	< .0001	46,696 (12.0%)	31,298 (15.8%)	< .0001
Dyspepsia or stomach discomfort	121,554 (20.7%)	157,020 (25.4%)	< .0001	78,200 (20.1%)	43,354 (21.9%)	< .0001
Peripheral vascular disease	274,992 (46.9%)	333,554 (54.0%)	< .0001	173,898 (44.7%)	101,094 (51.0%)	< .0001
Transient ischemic attack	47,167 (8.0%)	53,598 (8.7%)	< .0001	31,913 (8.2%)	15,254 (7.7%)	< .0001
Coronary artery disease	247,242 (42.1%)	294,580 (47.7%)	< .0001	158,793 (40.9%)	88,449 (44.6%)	< .0001
History of falls	42,554 (7.3%)	75,136 (12.2%)	< .0001	26,673 (6.9%)	15,881 (8.0%)	< .0001
Baseline medication usage ^a^						
ACE/ARB	348,128 (59.3%)	323,724 (52.4%)	< .0001	232,399 (59.8%)	115,729 (58.4%)	< .0001
Amiodarone	9044 (1.5%)	16,909 (2.7%)	< .0001	6118 (1.6%)	2926 (1.5%)	.0038
Beta blockers	282,705 (48.2%)	270,271 (43.8%)	< .0001	186,247 (47.9%)	96,458 (48.7%)	< .0001
H2-receptor antagonist	38,175 (6.5%)	51,268 (8.3%)	< .0001	24,545 (6.3%)	13,630 (6.9%)	< .0001
Proton pump inhibitor	174,744 (29.8%)	204,186 (33.1%)	< .0001	115,268 (29.7%)	59,476 (30.0%)	.0074
Statins	328,330 (55.9%)	315,799 (51.1%)	< .0001	218,810 (56.3%)	109,520 (55.2%)	< .0001
Anti-platelets	83,148 (14.2%)	103,992 (16.8%)	< .0001	54,377 (14.0%)	28,771 (14.5%)	< .0001
NSAIDS	142,065 (24.2%)	136,148 (22.0%)	< .0001	97,497 (25.1%)	44,568 (22.5%)	< .0001
Baseline all-cause health care utilization^†^						
Inpatient admission visit	298,336 (50.8%)	351,888 (57.0%)	< .0001	184,585 (47.5%)	113,751 (57.4%)	< .0001
ER visit	194,169 (33.1%)	234,104 (37.9%)	< .0001	125,059 (32.2%)	69,110 (34.9%)	< .0001
# of office visit (PPPM)	1.2 (1.1)	1.2 (1.2)	< .0001	1.2 (1.1)	1.2 (1.1)	< .0001

^**a**^Variables included in the multivariate logistic model

^b^As the INR value is not available in the databases, a modified HAS-BLED score was calculated with a range of 0 to 8

*ACE* angiotensin-converting enzyme inhibitors, *ARB* angiotensin receptor blockers, *DOAC* direct oral anticoagulants, *ER* emergency room, *NSAIDs* nonsteroidal anti-inflammatory drugs, *OAC* oral anticoagulants, *PPPM* per patient per month, *SD* standard deviation

## Results

### Baseline characteristics

After application of the selection criteria, 1,204,507 AF patients with a CHA_2_DS_2_-VASc ≥ 2 were identified. Out of these, 617,611 (51.3%) patients were not prescribed OAC therapy versus 586,896 (48.7%) patients who were prescribed OAC therapy (during follow-up).

**Fig. 2:**
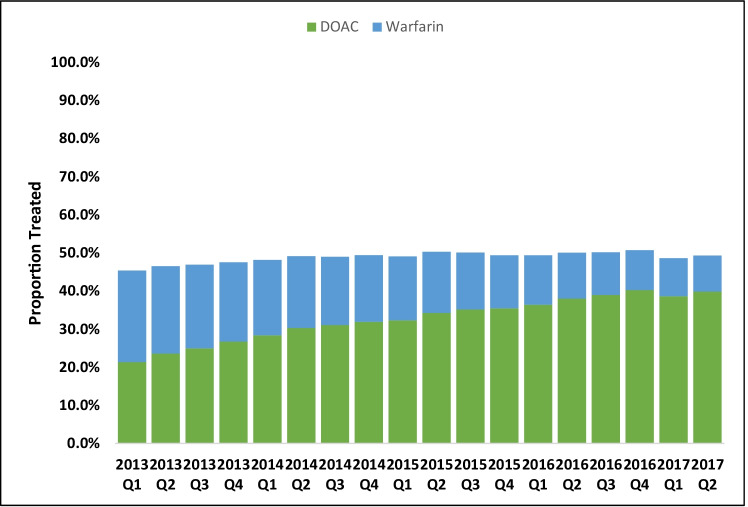
Trends of DOAC and warfarin prescription over time among incident AF Fee-for-Service Medicare Patients with CHA_2_DS_2_-VASc ≥ 2. AF, atrial fibrillation; DOAC, direct oral anticoagulants; Q1, first quarter; Q2, second quarter; Q3, third quarter; Q4, fourth quarter

Among those prescribed an OAC, 388,629 (66.2%) were prescribed a DOAC and 198,267 (33.8%) were prescribed warfarin. Patients who were prescribed DOACs were younger (77.8 ± 7.2 vs. 78.2 ± 7.4 years; *P* < 0.0001) and had lower CHA_2_DS_2_-VASc (4.4 ± 1.6 vs. 4.7 ± 1.6; *P* < 0.0001) and HAS-BLED (3.2 ± 1.2 vs. 3.4 ± 1.2; *P* < 0.0001) scores compared to patients who were prescribed warfarin (Table 1). Figure 2 shows the trend of OAC prescription with respect to DOAC and warfarin status for the duration of the study period. Throughout the study period, the proportion of patients with OAC underutilization was relatively consistent and remained above 50% throughout the study period. Of those patients prescribed OACs, the proportion of patients prescribed warfarin decreased from 52.8 to 19.2%, while the proportion of patients prescribed DOACs increased from 47.2 to 80.8% in incident AF patients during our study period (Fig. 2).

### Predictors of OAC therapy prescription

**Fig. 3:**
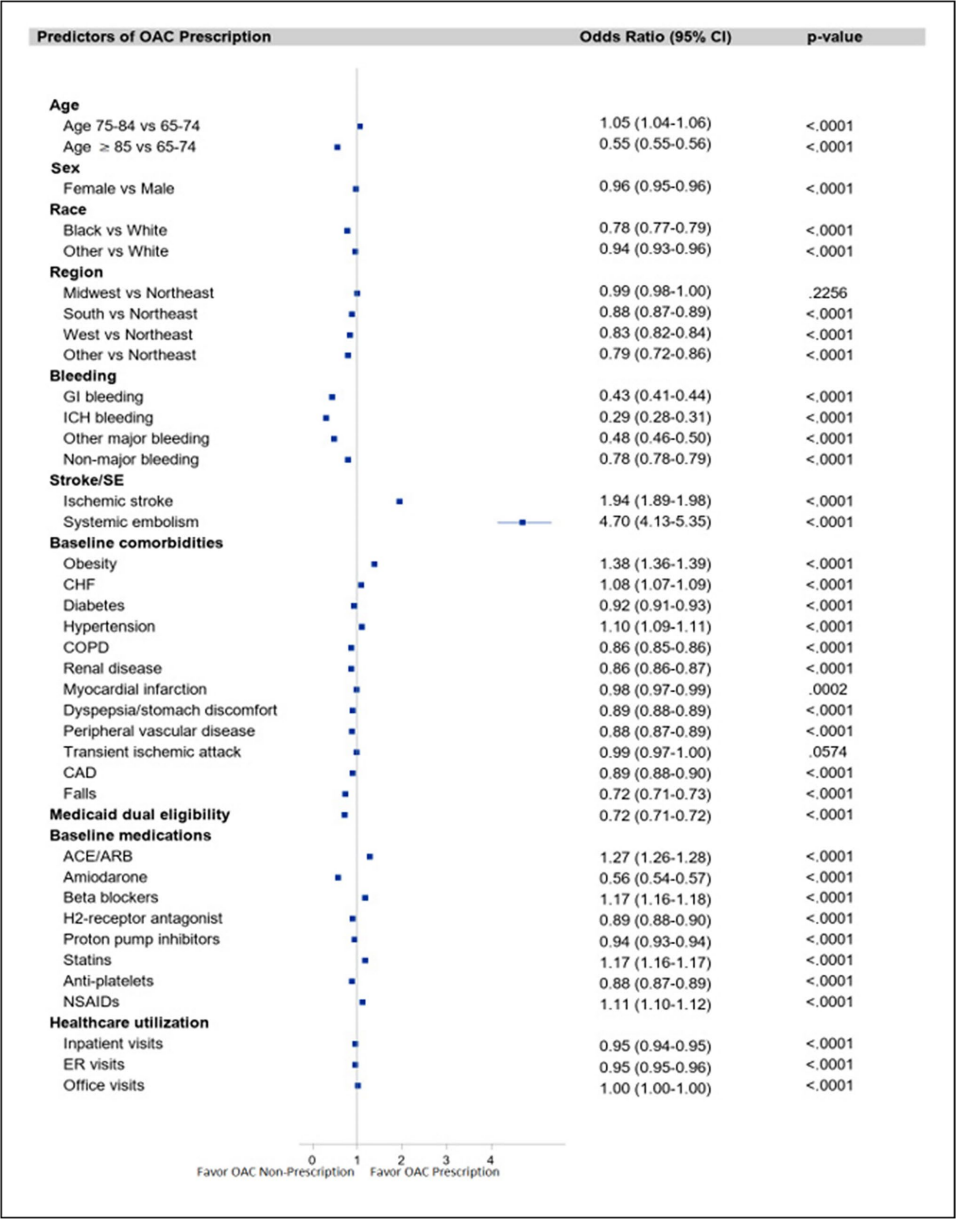
Predictors of OAC prescription vs No OAC prescription. ACE/ARB, angiotensin-converting enzyme inhibitors/angiotensin receptor blockers; CAD, coronary artery disease; CHF, congestive heart failure; CI, confidence interval; COPD, chronic obstructive pulmonary disease; ER, emergency room; ICH, intracranial hemorrhage; GI, gastrointestinal; NSAIDs, non-steroidal anti-inflammatory drugs; OAC, oral anticoagulants; SE, systemic embolism

The multivariable logistic regression model identified several significant predictors of OAC underutilization (Fig. 3). Age ≥ 85 years (OR 0.55, 95% CI 0.55–0.56), female sex (OR 0.96, 95% CI 0.95–0.96), Black race (OR 0.78, 95% CI 0.77–0.79), and key comorbidities such as coronary artery disease (OR 0.89, 95% CI 0.88–0.90), diabetes (OR 0.92, 95% CI 0.91–0.93), renal disease (OR 0.86, 95% CI 0.86–0.87), history of falls (OR 0.72, 95% CI 0.71–0.73), GI bleeding (OR 0.43, 95% CI 0.41–0.44), and intracranial bleeding (OR 0.29, 95% CI 0.28–0.31) were associated with underutilization of OAC therapy. Additionally, patients who were not prescribed OAC therapy were older (mean age 80.4 ± 8.7 vs 77.9 ± 7.3 years; *P* < 0.0001) and had higher CHA2DS2-VASc (4.8 ± 1.6 vs 4.5 ± 1.6; *P* < 0.0001) and HAS-BLED scores (3.5 ± 1.3 vs 3.3 ± 1.2; *P* < 0.0001) compared to those who were prescribed OAC therapy. A previous history of major bleeding (4.7% vs 1.6%; *P* < 0.0001) was more prevalent, while obesity (16.0% vs 21.6%; *P* < 0.0001) was less prevalent in those patients who were not prescribed an OAC compared to those prescribed an OAC (Table 1).Ischemic stroke (OR 1.94, 95% CI 1.89–1.98), SE (OR: 4.70, 95% CI 4.13–5.35), obesity (OR 1.38, 95% CI 1.36–1.39), congestive heart failure (OR 1.08, 95% CI 1.07–1.09), and hypertension (OR 1.10, 95% CI 1.09–1.1) were associated with higher odds of OAC prescription (Fig. 3). In a separate model, with age as a continuous variable, older patients were associated with a lower odds of OAC treatment (OR 0.97, 95% CI 0.97–0.97; Supplemental Table 2).

**Fig. 4:**
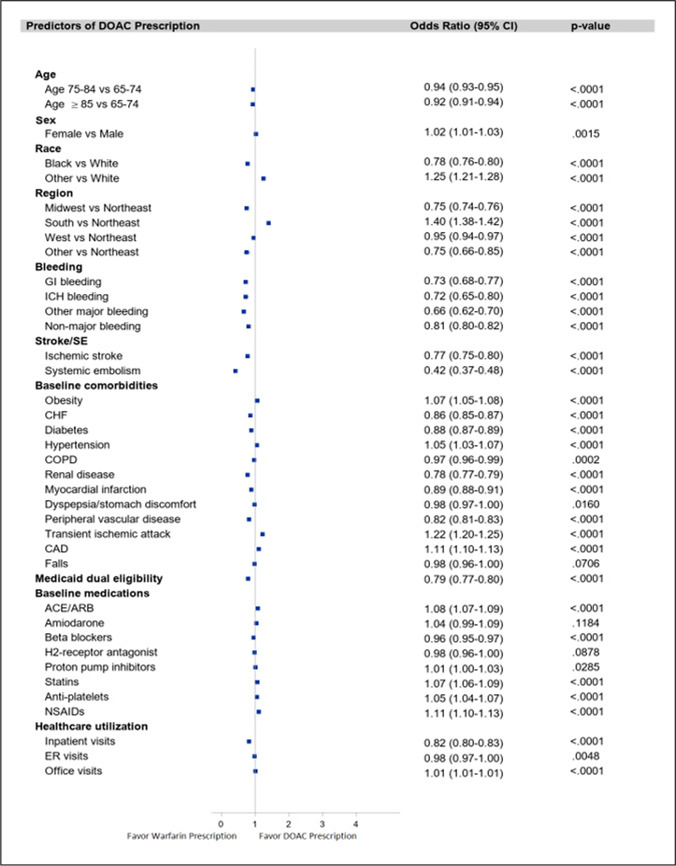
Predictors of DOAC versus warfarin prescription. ACE/ARB, angiotensin-converting enzyme inhibitors/angiotensin receptor blockers; CAD, coronary artery disease; CHF, congestive heart failure; CI, confidence interval; COPD, chronic obstructive pulmonary disease; ER, emergency room; ICH, intracranial hemorrhage; GI, gastrointestinal; NSAIDs, non-steroidal anti-inflammatory drugs; OAC, oral anticoagulants; SE, systemic embolism

Among AF patients prescribed an OAC, additional multivariable logistic models identified several significant predictors of DOAC versus warfarin prescription (Fig. 4). Characteristics that were associated with lower odds of DOAC versus warfarin prescription included: age ≥ 85 (OR 0.92, 95% CI 0.91–0.94), Black race (OR: 0.78, 95% CI 0.76–0.80), ischemic stroke (OR 0.77, 95% CI 0.75–0.80), GI bleeding (OR 0.73, 95% CI 0.68–0.77), intracranial bleeding (OR 0.72, 95% CI 0.65–0.80), residence in the Midwest region (OR 0.75, CI 0.74–0.76), and inpatient visits (OR 0.82, CI 0.80–0.83) (Fig. 4). Transient ischemic attack (OR 1.22, 95% CI 1.20–1.25), obesity (OR 1.07, 95% CI 1.05–1.08), and hypertension (OR 1.05, 95 CI 1.03–1.07) had higher odds of DOAC versus warfarin prescription.

Two prespecified sub-analyses describing the interaction of age were performed based on the overall full analysis. The first analysis evaluated the interaction of age with 3 prespecified patient-level variables on the outcome of OAC prescription versus no OAC prescription (Supplemental Table 3). The second analysis evaluated the interaction of age with 4 prespecified patient level variables on the outcome of DOAC versus warfarin prescription (Supplemental Table 4). For the first age interaction analysis, the 3 patient level variables of ischemic stroke, SE, and falls were identified a priori based on clinical knowledge and selected to examine effect modification of age on the association of patient level variables with OAC prescription. The odds of an OAC prescription significantly increased with advancing age in patients with a history of ischemic stroke (OR for ages 65–74 1.74, 95% CI 1.67–1.82; OR for ages 75–84 1.86 95% CI 1.79–1.93; OR for age ≥ 85 2.22, 95% CI 2.14–2.31). Similarly, the odds of an OAC prescription also significantly increased with advancing age in patients with a history of SE (OR for ages 65–74 3.13, 95% CI 2.53–3.88; OR for ages 75–84 4.24 95% CI 3.39–5.31; OR for age ≥ 85 7.61, 95% CI 6.03–9.60). On the contrary, the odds of an OAC prescription significantly decreased with advancing age and a prior history of falls (OR for age 65–74 0.80, 95% CI 0.78–0.82; OR for age 75–84 0.69, 95% CI 0.68–0.71; OR for age ≥ 85 0.71, 95% CI 0.70–0.73). For the second age interaction analysis, four patient level variables of ischemic stroke, SE, ICH, and renal disease were identified a priori based on clinical knowledge. These were selected to analyze effect modification of age on the association of patient level variables with DOAC versus warfarin prescription. In patients with a prior history of ICH, the odds of a DOAC prescription significantly increased across all age groups but warfarin was still most commonly prescribed (OR for ages 65–74 0.60, 95% CI 0.50–0.73; OR for ages 75–84 0.69 95% CI 0.60–0.81; OR for age ≥ 85 1.00, 95% CI 0.80–1.25). A similar trend in DOAC prescription was also observed in patients with renal disease, among whom the odds of a DOAC prescription significantly increased across all age groups; however, warfarin was still the preferred drug (OR for ages 65–74 0.67, 95% CI 0.65–0.68; OR for ages 75–84 0.80 95% CI 0.78–0.81; OR for age ≥ 85 0.91, 95% CI 0.89–0.94).

## Discussion

In this large real-world sample of Medicare patients ≥ 65 years old with a diagnosis of AF and stroke risk, we report several key findings. (1) The prevalence of OAC prescription continued to be low even after the introduction of DOACs in clinical practice, as underutilization remained common, with more than half of eligible patients in our study who were not prescribed an OAC during follow-up. (2) Over our study period, the proportion of patients prescribed warfarin reduced from 23.9 to 9.5%, and DOAC prescription increased from 21.4 to 39.9%, becoming the most commonly prescribed OAC. (3. Specific patient characteristics such as advanced age, female sex, Black race, and presence of important comorbidities (i.e., coronary artery disease, diabetes, renal disease, history of falls, and major bleeding) were associated with underutilization of OAC therapy. Moreover, most of the same variables predicted lower DOAC prescription among eligible AF patients. (4) There was an increase in odds of OAC (with preference for DOAC) prescription in AF patients with advancing age and history of ischemic stroke.

One of the most devastating complications of AF is ischemic stroke, and AF-related strokes tend to have worse mortality and morbidity when compared to strokes unrelated to AF [4, 5]. Before the introduction of DOACs, warfarin was the standard anticoagulant used to reduce stroke risk in eligible AF patients. Warfarin is characterized by unpredictable pharmacokinetics, extensive food and drug interactions, and frequent need for laboratory monitoring [16, 17]. Several earlier studies have shown underutilization of OAC therapy in eligible AF patients for stroke risk reduction. Most of these studies were done in the era when warfarin was still the standard of care for stroke prevention in AF patients, and such underutilization could be related to an unfavorable warfarin safety profile. In a systematic review conducted by Ogilvie et al. on AF patients with a prior history of stroke, the utilization of OAC (warfarin) therapy was only 60% [10]. Similarly, in a study of 9706 worldwide patients with AF, Suarez et al. showed warfarin utilization rate of 39.5% [13]. They also showed that only 28% of patients above 75 years of age were prescribed warfarin treatment. Additionally, a study of AF and congestive heart failure patients from the American Heart Association’s Get With the Guidelines Heart Failure program, Piccini et al. showed median prevalence of 64.9% for warfarin treatment (interquartile range 55.5–73.4) among eligible patients [11]. In a more contemporary analysis of AF patients insured by private companies, Al-Khatib et al. demonstrated that nearly one-third of such patients were not treated with an OAC [9]. Their study period encompassed the time frame in which DOACs were assimilated in clinical practice. Our more contemporary study of nearly 1.2 million Medicare patients ≥ 65 years old with AF showed a gradual trend towards increase in DOAC prescription (which has become the more commonly prescribed OAC) but overall prevalence of OAC prescription continued to be below guideline-based recommendation in our cohort of patients with elevated stroke risk (CHA_2_DS_2_-VASc score ≥ 2) (Fig. 2). Of note, our findings indicated lower utilization of OACs among patients with a higher CHA_2_DS_2_-VASc score and this may be associated with a higher burden of comorbidities in our elderly cohort of AF patients. Additionally, advanced age (one of the important components of CHA2DS2-VASc) increases the propensity for mechanical falls with a subsequent significant bleeding event which may also explain the lower utilization of OACs in our study group.

Due to the low prevalence of OAC prescription in eligible AF patients, it is imperative to assess specific patient characteristics that are associated with OAC underutilization. In our study, advanced age (≥ 85 years), female sex, Black race, and comorbidities such as coronary artery disease, diabetes, renal disease, history of falls, GI bleeding, and intracranial bleeding predicted underutilization of OAC therapy. In a study of 674,841 AF patients who met criteria for anti-coagulation from the National Cardiovascular Data Registry (NCDR) Pinnacle registry, Lubitz et al. [14] also demonstrated that female sex and renal disease predicted under-prescription of OAC therapy. In a Veterans Health Administration study among 10,212 device-detected AF patients stratified by length of AF episode, Perino et al. [18] found low levels of OAC prescription overall, even among patients with AF episodes lasting ≥ 24 h (224 of 818 patients were prescribed an OAC after an AF episode ≥ 24 h). In another study from the NCDR Pinnacle registry, Thompson et al. [19] showed that OACs were underutilized in women as compared to men (56.7% vs. 61.3%, *p* < 0.001). This lower utilization in women persisted at all levels of CHA_2_DS_2_-VASc score. The etiology behind this low utilization of OAC therapy in women is unclear but could be related to both patient and provider preference. Shantsila et al. [20] reported that women are more likely to refuse OAC due to bleeding concerns and lack of logistic support required for frequent laboratory monitoring, especially if they are prescribed warfarin; however, other societal and environmental factors may be at play. Indeed, our study has shown increased odds of DOAC prescription in women compared to men (OR 1.02, 95% CI 1.01–1.03) perhaps due to the aforementioned reason. There may be a bias on the part of providers in applying relevant guidelines to the female population, which has also contributed to low OAC prescription prevalence [21, 22]. Similarly, the low prevalence of OAC prescription in patients with renal disease could be related to perceived risk of bleeding in such patients. In our study, patients with renal disease also have lower odds of DOAC prescription when compared to warfarin (OR 0.78, 95% CI 0.77–0.79). In a recent meta-analysis of 34,082 AF patients with mild to moderate chronic kidney disease, Ha et al. [23] demonstrated no increased risk of bleeding with DOAC utilization (relative risk [RR] for major bleeding 0.80, 95% CI 0.61–1.04; RR for ICH 0.49, 95% CI 0.30–0.80), indicating that DOACs can be safely utilized in such patients. Our study also showed that Black race was associated with lower utilization of OAC and DOAC prescription therapy. In a study conducted by Essien et al. with 12,417 AF patients, Black patients were less likely than White patients to receive DOAC therapy, even after controlling for various clinical and socioeconomic factors (adjusted OR 0.63, 95% CI 0.49–0.83) [24]. Additional studies informing on clinical, demographic, and socioeconomic factors are needed to address such disparities in Black patients. Our study also showed lower odds of OAC prescription in patients with coronary artery disease. This low prevalence can be attributed to increased bleeding risk in such patients, as they are often concomitantly prescribed anti-platelet therapy, although recent studies have shown reduction in bleeding risk in these patients when dual therapy (DOAC with either aspirin or a P2Y12 inhibitor) was utilized instead of triple therapy [25]. Additionally, earlier studies have also demonstrated improved utilization of OAC therapy in eligible AF patients with the implementation of structured educational programs. In a large, randomized IMPACT-AF (a multifaceted intervention to improve treatment with oral anticoagulants in atrial fibrillation: an international, cluster-randomized trial) study, Vinereanu et al. have demonstrated that a multiprong educational intervention aimed at both patients and physicians resulted in improved utilization of OAC from 68% at baseline to approximately 80% at 1 year of follow-up in the intervention group (OR 3.28, 95% CI 1.67–6.44) [26].

Our age interaction analysis showed increased odds of OAC prescription in AF patients with history of ischemic stroke with advancing age. Similarly, we also demonstrated increased odds of DOAC prescription in AF patients with prior history of ischemic stroke as they aged. In a study of 8932 patients, van Walraven et al. showed increased risk of ischemic stroke with patients age (adjusted hazard ratio per decade of age increase 1.45, 95% CI 1.26–1.66) [6]. They also demonstrated that as these patients get older, the absolute benefit of OAC in reducing the incidence of ischemic stroke increases. Moreover, while clinical guidelines recommend DOACs over warfarin for certain older patients, and uptake appears to be moving clinical practice toward more utilization of DOACs in these groups, greater guideline awareness and adherence may help address persistent gaps between evidence and practice. In this context, our study findings provide further evidence that advanced age should not be the only contraindication in prescribing an OAC for stroke risk mitigation; practice patterns appear to align with these important findings.

## Strengths and limitations

The primary strengths of this study are the large sample size, the long follow-up period, and sufficient statistical power necessary to assess significant predictors of OAC underutilization as well as predictors of DOAC versus warfarin prescription. Our sample includes a nationally representative aging population, as nearly two-thirds of Americans aged ≥ 65 years are enrolled in a fee-for-service Medicare health plan.

This study has some limitations that should be considered while interpreting the results. This is a retrospective observational study and thus causal inference cannot be evaluated. The selected variables were based on ICD-9-CM diagnosis and procedure codes, the Healthcare Common Procedure Coding System, and National Drug Codes on billing claims. As such, coding errors and lack of clinical accuracy may have introduced bias in the study. In fact, early studies have reported up to 30% false positive and inactive AF patients when extracted from large scale registries and elimination of such cases have shown to improve the OAC utilization [27]. Additionally, differential follow-up of AF patients in our cohort may have introduced selection bias, as patients with longer follow-ups were more likely to be treated with an OAC. In addition, the Medicare database does not include laboratory values or self-reported data, thus outcomes and risk assessment (such as the modified HAS-BLED score) should be interpreted in the context of coding algorithms. Changes in patient characteristics between diagnosis and prescription were not accounted for in the logistic regression models. Presence of prescription claims may not indicate that the medication was consumed by the patient. In addition, the covariates may have been impacted by heterogeneity. More research is needed to understand the importance of each covariate along with validation. Our study also did not consider drug costs, access to care, formulary changes, or physician type or preferences. In addition, the study results may not be generalizable to the entire US population, as this study only observed incident AF patients from Medicare fee-for-service data, which only includes patients ≥ 65 years old and certain special groups of patients. Similarly, dual Veterans Health Administration beneficiary status was not observable in the dataset, and dual beneficiaries may have contributed to under- or overestimation of utilization.

## Conclusion

This contemporary real-world study of Medicare patients ≥ 65 years old with AF and a CHA_2_DS_2_-VASc ≥ 2 found that OAC utilization is still low among older US patients. Several key predictors of OAC underutilization were identified, including age ≥ 85 years, female sex, Black race, and key comorbidities such as coronary artery disease, diabetes, renal disease, history of falls, and GI and intracranial bleeding. Furthermore, age ≥ 85 years, Black race, ischemic stroke, and GI and intracranial bleeding predicted lower use of DOAC therapy compared to warfarin.

## Supplementary Information

Below is the link to the electronic supplementary material.

Supplementary file1 (DOCX 51 KB)

## Data Availability

The raw insurance claims data used for this study originate from Medicare data, which are available from the Centers for Medicare and Medicaid through ResDAC (https://www.resdac.org/). Other researchers could access the data through ResDAC, and the inclusion criteria specified in the Methods section would allow them to identify the same cohort of patients we used for these analyses.
