# Supplementary material for: Oral anticoagulant underutilization among elderly patients with atrial fibrillation: insights from the United States Medicare database
Source: J Interv Card Electrophysiol. 2022 Jul 9;66(3):771–82. doi: 10.1007/s10840-022-01274-1 (PMC10066118; doi:10.1007/s10840-022-01274-1)
Supplement: Supplementary file 1 — Supplementary file1 (DOCX 51 KB) [file 10840_2022_1274_MOESM1_ESM.docx]

**Supplemental Tables**

**Supplemental Table 1. Codes Used for Identifying Diagnoses/Conditions Used in the Study**

| **Exclusion Criteria Diagnoses** | | |
| --- | --- | --- |
| **Diagnosis/Category** | **ICD-9-CM Codes** | **ICD-10-CM Codes** |
| Valvular Heart Disease | 394.0, 394.1, 394.2, 394.9, 396.0, 396.1, 396.8, 396.9, 424.0, 745.xx | I050, I051, I052, I058, I059, I080, I088, I089, I340, I341, I342, I348, I349, Q213, Z952, Z953, Z954 |
| Valve Replacement Procedure | 35.05-35.09, 35.20-35.28, 35.97 | 02RF07Z, 02RF08Z, 02RF0JZ, 02RF0KZ, 02RF37H, 02RF37Z, 02RF38H, 02RF38Z, 02RF3JH, 02RF3JZ, 02RF3KH, 02RF3KZ, 02RF47Z, 02RF48Z, 02RF4JZ, 02RF4KZ, 02RG07Z, 02RG08Z, 02RG0JZ, 02RG0KZ, 02RG37H, 02RG37Z, 02RG38H, 02RG38Z, 02RG3JH, 02RG3JZ, 02RG3KH, 02RG3KZ, 02RG47Z, 02RG48Z, 02RG4JZ, 02RG4KZ, 02RH07Z, 02RH08Z, 02RH0JZ, 02RH0KZ, 02RH37H, 02RH37Z, 02RH38H, 02RH38Z, 02RH3JH, 02RH3JZ, 02RH3KH, 02RH3KZ, 02RH47Z, 02RH48Z, 02RH4JZ, 02RH4KZ, 02RJ07Z, 02RJ08Z, 02RJ0JZ, 02RJ0KZ, 02RJ47Z, 02RJ48Z, 02RJ4JZ, 02RJ4KZ, 02UG3JZ, X2RF032, X2RF332, X2RF432 |
| **CHA2DS2-VASC SCORE CODES** | | |
| Congestive Heart Failure | 402.x1, 404.x1, 404.x3, 428.xx | I0981, I110, I132, I501, I5020, I5021, I5022, I5023, I5030, I5031, I5032, I5033, I5040, I5041, I5042, I5043, I509 |
| Hypertension | 401.xx-405.xx | I10, I110, I119, I120, I129, I130, I1310, I1311, I132, I150, I151, I152, I158, I159, I160, I161, I169, N262 |
| Age≥ 75 years | - | - |
| Diabetes mellitus | 250, 357.2, 362.0, 366.41 | E08-E13 |
| Stroke (non-hemorrhagic only & transient ischemic attack) | V12.54, 433.xx-436.xx | I63, I65, I66 |
| Vascular Disease (myocardial infarction, peripheral arterial disease, aortic plaque) | 410.xx, 412, 440.xx, 441.xx, 442.xx, 443.xx, 444.2x, 445.0x | I200, I2101, I2102, I2109, I2111, I2119, I2121, I2129, I213, I214, I220, I221, I222, I228, I229, I240, I241, I248, I249, I25110, I252, I25700, I25710, I25720, I25730, I25750, I25760, I25790, I670, I700, I701, I70201, I70202, I70203, I70208, I70209, I70211, I70212, I70213, I70218, I70219, I70221, I70222, I70223, I70228, I70229, I70231, I70232, I70233, I70234, I70235, I70238, I70239, I70241, I70242, I70243, I70244, I70245, I70248, I70249, I7025, I70261, I70262, I70263, I70268, I70269, I70291, I70292, I70293, I70298, I70299, I70301, I70302, I70303, I70308, I70309, I70311, I70312, I70313, I70318, I70319, I70321, I70322, I70323, I70328, I70329, I70331, I70332, I70333, I70334, I70335, I70338, I70339, I70341, I70342, I70343, I70344, I70345, I70348, I70349, I7035, I70361, I70362, I70363, I70368, I70369, I70391, I70392, I70393, I70398, I70399, I70401, I70402, I70403, I70408, I70409, I70411, I70412, I70413, I70418, I70419, I70421, I70422, I70423, I70428, I70429, I70431, I70432, I70433, I70434, I70435, I70438, I70439, I70441, I70442, I70443, I70444, I70445, I70448, I70449, I7045, I70461, I70462, I70463, I70468, I70469, I70491, I70492, I70493, I70498, I70499, I70501, I70502, I70503, I70508, I70509, I70511, I70512, I70513, I70518, I70519, I70521, I70522, I70523, I70528, I70529, I70531, I70532, I70533, I70534, I70535, I70538, I70539, I70541, I70542, I70543, I70544, I70545, I70548, I70549, I7055, I70561, I70562, I70563, I70568, I70569, I70591, I70592, I70593, I70598, I70599, I70601, I70602, I70603, I70608, I70609, I70611, I70612, I70613, I70618, I70619, I70621, I70622, I70623, I70628, I70629, I70631, I70632, I70633, I70634, I70635, I70638, I70639, I70641, I70642, I70643, I70644, I70645, I70648, I70649, I7065, I70661, I70662, I70663, I70668, I70669, I70691, I70692, I70693, I70698, I70699, I70701, I70702, I70703, I70708, I70709, I70711, I70712, I70713, I70718, I70719, I70721, I70722, I70723, I70728, I70729, I70731, I70732, I70733, I70734, I70735, I70738, I70739, I70741, I70742, I70743, I70744, I70745, I70748, I70749, I7075, I70761, I70762, I70763, I70768, I70769, I70791, I70792, I70793, I70798, I70799, I708, I7090, I7091, I7092, I7100, I7101, I7102, I7103, I711, I712, I713, I714, I715, I716, I718, I719, I720, I721, I722, I723, I724, I725, I726, I728, I729, I7300, I7301, I731, I7381, I7389, I739, I742, I743, I744, I75011, I75012, I75013, I75019, I75021, I75022, I75023, I75029, I7770, I7771, I7772, I7773, I7774, I7775, I7776, I7777, I7779, I790, I791, I798 |
| Age | Age 65-75 years | - |
| Sex Category | - | - |
| **HAS-BLED SCORE CODES** | | |
| Hypertension | 401.xx-405.xx | I10, I110, I119, I120, I129, I130, I1310, I1311, I132, I150, I151, I152, I158, I159, I160, I161, I169, N262 |
| Abnormal kidney and/or liver function: | Kidney: 580.xx-589.xx | B520, E0821, E0822, E0829, E0921, E0922, E0929, M3214, M3215, M3504, N00-N08, N140, N141, N142, N143, N144, N150, N158, N159, N16, N170, N171, N172, N178, N179, N181, N182, N183, N184, N185, N186, N189, N19, N250, N251, N2581, N2589, N259, N261, N269, N270, N271, N279 |
|  | Liver: 570.xx-573.xx | B251, K70-K77 |
| Stroke | History of stroke V12.54, 433.xx-436.xx | G450, G451, G452, G458, G459, G460, G461, G462, I63, I65, I66, I67841, I67848, Z8673 |
| Bleeding | 280.xx-286.xx | A985, D500, D508, D509, D510, D511, D512, D513, D518, D519, D520, D521, D528, D529, D530, D531, D532, D538, D539, D550, D551, D552, D553, D558, D559, D560, D561, D562, D563, D565, D568, D569, D5700, D5701, D5702, D571, D5720, D57211, D57212, D57219, D573, D5740, D57411, D57412, D57419, D5780, D57811, D57812, D57819, D580, D581, D582, D588, D589, D590, D591, D592, D593, D594, D595, D596, D598, D599, D600, D601, D608, D609, D6101, D6109, D611, D612, D613, D61810, D61811, D61818, D6182, D6189, D619, D62, D62, D630, D631, D638, D640, D641, D642, D643, D644, D6481, D6489, D649, D65, D66, D67, D680, D681, D682, D68311, D68312, D68318, D6832, D684, D688, D689, D7801, D7802, D7821, D7822, E3601, E3602, E89810, E89811, G9731, G9732, G9751, G9752, H05231, H05232, H05233, H05239, H1130, H1131, H1132, H1133, H2100, H2101, H2102, H2103, H31301, H31302, H31303, H31309, H31311, H31312, H31313, H31319, H31411, H31412, H31413, H31419, H3560, H3561, H3562, H3563, H35731, H35732, H35733, H35739, H4310, H4311, H4312, H4313, H44811, H44812, H44813, H44819, H47021, H47022, H47023, H47029, H59111, H59112, H59113, H59119, H59121, H59122, H59123, H59129, H59311, H59312, H59313, H59319, H59321, H59322, H59323, H59329, H61121, H61122, H61123, H61129, H9521, H9522, H9541, H9542, I312, I6000, I6001, I6002, I6010, I6011, I6012, I602, I6030, I6031, I6032, I604, I6050, I6051, I6052, I606, I607, I608, I609, I610, I611, I612, I613, I614, I615, I616, I618, I619, I6200, I6201, I6202, I6203, I621, I629, I8501, I8511, I97410, I97411, I97418, I9742, I97610, I97611, I97618, I97620, J9561, J9562, J95830, J95831, K2211, K226, K250, K252, K254, K256, K260, K262, K264, K266, K270, K272, K274, K276, K280, K282, K284, K286, K2901, K2921, K2931, K2941, K2951, K2961, K2971, K2981, K2991, K31811, K3182, K5521, K5701, K5711, K5713, K5721, K5731, K5733, K5741, K5751, K5753, K5781, K5791, K5793, K625, K6381, K640, K641, K642, K643, K648, K661, K9161, K9162, K91840, K91841, K920, K921, K922, L7601, L7602, L7621, L7622, M2500, M25011, M25012, M25019, M25021, M25022, M25029, M25031, M25032, M25039, M25041, M25042, M25049, M25051, M25052, M25059, M25061, M25062, M25069, M25071, M25072, M25073, M25074, M25075, M25076, M2508, M96810, M96811, M96830, M96831, N421, N837, N857, N920, N9961, N9962, N99820, N99821, R040, R041, R042, R0489, R049, R310, R319, R58, R710, S0190XA, S062X0A, S062X1A, S062X2A, S062X3A, S062X4A, S062X5A, S062X6A, S062X7A, S062X8A, S062X9A, S06300A, S06301A, S06302A, S06303A, S06304A, S06305A, S06306A, S06307A, S06308A, S06309A, S06340A, S06341A, S06342A, S06343A, S06344A, S06345A, S06346A, S06347A, S06348A, S06349A, S06350A, S06351A, S06352A, S06353A, S06354A, S06355A, S06356A, S06357A, S06358A, S06359A, S06360A, S06361A, S06362A, S06363A, S06364A, S06365A, S06366A, S06367A, S06368A, S06369A, S064X0A, S064X1A, S064X2A, S064X3A, S064X4A, S064X5A, S064X6A, S064X7A, S064X8A, S064X9A, S065X0A, S065X1A, S065X2A, S065X3A, S065X4A, S065X5A, S065X6A, S065X7A, S065X8A, S065X9A, S066X0A, S066X1A, S066X2A, S066X3A, S066X4A, S066X5A, S066X6A, S066X7A, S066X8A, S066X9A, S06890A, S06891A, S06892A, S06893A, S06894A, S06895A, S06896A, S06897A, S06898A, S06899A, S069X0A, S069X1A, S069X2A, S069X3A, S069X4A, S069X5A, S069X6A, S069X7A, S069X8A, S069X9A, S36112A, S37021A, S37022A, S37029A, T792XXA |
| Labile INR | Not measurable. | Not applicable |
| Elderly | 65+ years | 65+ years |
| Alcohol/ Drug Therapy | 303.xx, 305.0x, V11.3x  Antiplatelets administered (abciximab, anagrelide HCL, aspirin, aspirin/dipyridamole, cilostazol, clopidogrel, dipyridamole, eptifibatide, prasugrel, ticagrelor, ticlopidine, tirofiban) NSAIDs administered (bromfenac, celecoxib, diclofenac, etodolac, fenoprofen, flurbiprofen, ibuprofen, indomethacin, ketoprofen, ketorolac, lansoprazole/naproxen, meclofenamate, mefenamic acid, meloxicam, nabumetone, naproxen, oxaprozin, piroxicam, sulindac, tolmetin) | F1010, F10120, F10129, F1020, F1021, F10220, F10229 |
| **Baseline Covariates** | | |
| Non-major Bleed (Claims which are not both inpatient and at primary diagnosis position) | **Gastrointestinal**: 455.2, 455.5, 455.8, 456.0x, 456.2x, 459.0x, 530.21, 530.7x, 530.82, 531.0x, 531.2x, 531.4x, 531.6x, 532.0x, 532.2x, 532.4x, 532.6x, 533.0x, 533.2x, 533.4x, 533.6x, 534.0x, 534.2x, 534.4x, 534.6x, 535.01, 535.11, 535.21, 535.31, 535.41, 535.51, 535.61, 535.71, 537.83, 537.84, 562.02, 562.03, 562.12, 562.13, 568.81, 569.3x, 569.85, 569.86, 578.xx, 784.8 (CPT: 43227, 43255, 43501, 44366, 44378, 44391, 45317, 45334, 45382, 46614; ICD-9 procedure: 44.43, 44.44, 44.49) **Intracranial**: 430.xx, 431.xx, 432..xx, 852.xx, 853.xx, 854.xx  **Other**: Blood Transfusion: (HCPCS codes - C1010, C1016, C1018, C1020, C1021, C9504, C9505, P9010, P9011, P9016, P9021, P9022, P9038, P9039, P9040, P9051, P9054, P9056, P9057, P9058, S3906; ICD-9 procedure: 99.03, 99.04; Facility revenue codes: 381, 382) 336.1, 360.43, 362.43, 362.81, 363.72, 364.41, 363.61, 363.62, 377.42, 379.23, 423.x, 719.1x, 997.02, (CPT: 32658, 33020, 47350-47362, 65815, 65930) 285.1, 376.32, 459.0x, 568.81, 573.8, 593.81, 596.7x, 599.7x, 790.01, 864.01, 865.01, 866.01, 958.2, 998.11 078.6, 246.3, 372.72, 374.81, 380.31, 602.1, 620.7, 621.4x, 623.6, 626.2, 640.xx, 641.9x, 666.10, 784.7x , 784.8x, 786.3 CPT codes: 30901, 30903, 30905, 31238, 42970; ICD-9 procedure codes: 21.01, 21.07 | I8501, I8511, K2211, K226, K250, K252, K254, K256, K260, K262, K264, K266, K270, K272, K274, K276, K280, K282, K284, K286, K2901, K2921, K2931, K2941, K2951, K2961, K2971, K2981, K2991, K31811, K3182, K5521, K5701, K5711, K5713, K5721, K5731, K5733, K5741, K5751, K5753, K5781, K5791, K5793, K625, K6381, K661, K920, K921, K922, R041, R58, I6000, I6001, I6002, I6010, I6011, I6012, I602, I6030, I6031, I6032, I604, I6050, I6051, I6052, I606, I607, I608, I609, I610, I611, I612, I613, I614, I615, I616, I618, I619, I6200, I6201, I6202, I6203, I621, I629, S0190XA, S062X0A, S062X1A, S062X2A, S062X3A, S062X4A, S062X5A, S062X6A, S062X7A, S062X8A, S062X9A, S06300A, S06301A, S06302A, S06303A, S06304A, S06305A, S06306A, S06307A, S06308A, S06309A, S06340A, S06341A, S06342A, S06343A, S06344A, S06345A, S06346A, S06347A, S06348A, S06349A, S06350A, S06351A, S06352A, S06353A, S06354A, S06355A, S06356A, S06357A, S06358A, S06359A, S06360A, S06361A, S06362A, S06363A, S06364A, S06365A, S06366A, S06367A, S06368A, S06369A, S064X0A, S064X1A, S064X2A, S064X3A, S064X4A, S064X5A, S064X6A, S064X7A, S064X8A, S064X9A, S065X0A, S065X1A, S065X2A, S065X3A, S065X4A, S065X5A, S065X6A, S065X7A, S065X8A, S065X9A, S066X0A, S066X1A, S066X2A, S066X3A, S066X4A, S066X5A, S066X6A, S066X7A, S066X8A, S066X9A, S06890A, S06891A, S06892A, S06893A, S06894A, S06895A, S06896A, S06897A, S06898A, S06899A, S069X0A, S069X1A, S069X2A, S069X3A, S069X4A, S069X5A, S069X6A, S069X7A, S069X8A, S069X9A, A985, D62, D7801, D7802, D7821, D7822, E3601, E3602, E89810, E89811, G9731, G9732, G9751, G9752, H05231, H05232, H05233, H05239, H1130, H1131, H1132, H1133, H2100, H2101, H2102, H2103, H31301, H31302, H31303, H31309, H31311, H31312, H31313, H31319, H31411, H31412, H31413, H31419, H3560, H3561, H3562, H3563, H35731, H35732, H35733, H35739, H4310, H4311, H4312, H4313, H44811, H44812, H44813, H44819, H47021, H47022, H47023, H47029, H59111, H59112, H59113, H59119, H59121, H59122, H59123, H59129, H59311, H59312, H59313, H59319, H59321, H59322, H59323, H59329, H61121, H61122, H61123, H61129, H9521, H9522, H9541, H9542, I312, I97410, I97411, I97418, I9742, I97610, I97611, I97618, I97620, J9561, J9562, J95830, J95831, K661, K9161, K9162, K91840, K91841, L7601, L7602, L7621, L7622, M2500, M25011, M25012, M25019, M25021, M25022, M25029, M25031, M25032, M25039, M25041, M25042, M25049, M25051, M25052, M25059, M25061, M25062, M25069, M25071, M25072, M25073, M25074, M25075, M25076, M2508, M96810, M96811, M96830, M96831, N421, N837, N857, N920, N9961, N9962, N99820, N99821, R040, R041, R042, R0489, R049, R310, R319, R58, R710, S36112A, S37021A, S37022A, S37029A, T792XXA Procedure codes: 43227, 43255, 43501, 44366, 44378, 44391, 45317, 45334, 45382, 46614, P9010, P9011, P9016, P9021, P9022, P9038, P9039, P9040, P9051, P9054, P9056, P9057, P9058, 30901, 30903, 30905, 31238, 32658, 33020, 42970, 47350, 47360, 47361, 47362, 65815, 65930, 04L23DZ, 06L13DZ, 06L43DZ, 06L53DZ, 0DQ60ZZ, 0DQ63ZZ, 0DQ64ZZ, 0DQ67ZZ, 0DQ68ZZ, 0DQ90ZZ, 0DQ93ZZ, 0DQ94ZZ, 0DQ97ZZ, 0DQ98ZZ, 0W3G0ZZ, 0W3G3ZZ, 0W3G4ZZ, 0W3P8ZZ, 2Y41X5Z, 30230H1, 30230N1, 30230P1, 30233H1, 30233N1, 30233P1, 30240H1, 30240N1, 30240P1, 30243H1, 30243N1, 30243P1, 30250H1, 30250N1, 30250P1, 30253H1, 30253N1, 30253P1, 30260H1, 30260N1, 30260P1, 30263H1, 30263N1, 30263P1 |
| Obesity | 278.0, 278.00, 278.01, 278.03, V85.3, V85.30, V85.31, V85.32, V85.33, V85.34, V85.35, V85.36, V85.37, V85.38, V85.39, V85.4, V85.41, V85.42, V85.43, V85.44, V85.45 (any DX on the claim) | E66.01, E66.09, E66.1, E66.2, E66.8, E66.9, Z68.30, Z68.31, Z68.32, Z68.33, Z68.34, Z68.35, Z68.36, Z68.37, Z68.38, Z68.39, Z68.41, Z68.42, Z68.43, Z68.44, Z68.45 |
| Congestive Heart Failure (CHF) | 398.91, 402.x1, 404.x3, 428.xx | I0981, I110, I132, I501, I5020, I5021, I5022, I5023, I5030, I5031, I5032, I5033, I5040, I5041, I5042, I5043, I509 |
| Diabetes Mellitus | 250.xx, 357.2, 362.0, 366.41 | E08-E13 |
| Hypertension | 401.xx -405.xx | I10, I110, I119, I120, I129, I130, I1310, I1311, I132, I150, I151, I152, I158, I159, I160, I161, I169, N262 |
| COPD | 491, 492, or 496 | J41-J44 |
| Renal Disease | 582.xx; 583.0x-583.79; 585.xx-586.99; 588.xx | B520, E0821, E0822, E0829, E0921, E0922, E0929, I120, I129, I130, I1310, I1311, I132, M3214, M3215, M3504, N000, N001, N002, N003, N004, N005, N006, N007, N008, N009, N010, N011, N012, N013, N014, N015, N016, N017, N018, N019, N020, N021, N022, N023, N024, N025, N026, N027, N028, N029, N030, N031, N032, N033, N034, N035, N036, N037, N038, N039, N040, N041, N042, N043, N044, N045, N046, N047, N048, N049, N050, N051, N052, N053, N054, N055, N056, N057, N058, N059, N060, N061, N062, N063, N064, N065, N066, N067, N068, N069, N070, N071, N072, N073, N074, N075, N076, N077, N078, N079, N08, N140, N141, N142, N143, N144, N150, N158, N159, N16, N170, N171, N172, N178, N179, N181, N182, N183, N184, N185, N186, N189, N19, Z992, Z9115, Z4931, Z4901, Z4902, Z4931, Z4932, Z4932 Procedure codes: 5A1D00Z, 5A1D60Z, 05HY33Z, 06HY33Z, 03130ZD, 03140ZD, 03150ZD, 03160ZD, 03170ZD, 03180ZD, 03190ZF, 031A0ZF, 031B0ZF, 031C0ZF, 031209D, 031209F, 03120AD, 03120AF, 03120JD, 03120JF, 03120KD, 03120KF, 03120ZD, 03120ZF, 031309D, 031309F, 03130AD, 03130AF, 03130JD, 03130JF, 03130KD, 03130KF, 03130ZD, 03130ZF, 031409D, 031409F, 03140AD, 03140AF, 03140JD, 03140JF, 03140KD, 03140KF, 03140ZD, 03140ZF, 031509D, 031509F, 03150AD, 03150AF, 03150JD, 03150JF, 03150KD, 03150KF, 03150ZD, 03150ZF, 031609D, 031609F, 03160AD, 03160AF, 03160JD, 03160JF, 03160KD, 03160KF, 03160ZD, 03160ZF, 031709D, 031709F, 03170AD, 03170AF, 03170JD, 03170JF, 03170KD, 03170KF, 03170ZD, 03170ZF, 031809D, 031809F, 03180AD, 03180AF, 03180JD, 03180JF, 03180KD, 03180KF, 03180ZD, 03180ZF, 031909F, 03190AF, 03190JF, 03190KF, 03190ZF, 031A09F, 031A0AF, 031A0JF, 031A0KF, 031A0ZF, 031B09F, 031B0AF, 031B0JF, 031B0KF, 031B0ZF, 031C09F, 031C0AF, 031C0JF, 031C0KF, 031C0ZF, 03PY07Z, 03PY0JZ, 03PY0KZ, 03PY37Z, 03PY3JZ, 03PY3KZ, 03PY47Z, 03PY4JZ, 03PY4KZ, 03130JD, 03140JD, 03150JD, 03160JD, 03170JD, 03180JD, 03190JF, 031A0JF, 031B0JF, 031C0JF, 3E1M39Z |
| Myocardial Infarction | 410.xx, 412.xx | I2101, I2102, I2109, I2111, I2119, I2121, I2129, I213, I214, I220, I221, I222, I228, I229, I252 |
| Dyspepsia or Stomach Discomfort | 787.1, 789.0, 789.4, 789.6, 536.8 | R100, R1010-R1012, K30, R1013, R102, R10811, R10812, R10813, R10814, R10816, R10817, R10819, R10821, R10822, R10826, R10827, R10829, R1084, R109, R12, R1930, R1931, R1932, R1936, R1937 |
| Peripheral artery disease | 412.xx-414.xx, 440.xx-445.xx | I200, I2101, I2102, I2109, I2111, I2119, I2121, I2129, I213, I214, I220, I221, I222, I228, I229, I240, I241, I248, I249, I25110, I252, I25700, I25710, I25720, I25730, I25750, I25760, I25790, I670, I700, I701, I70201, I70202, I70203, I70208, I70209, I70211, I70212, I70213, I70218, I70219, I70221, I70222, I70223, I70228, I70229, I70231, I70232, I70233, I70234, I70235, I70238, I70239, I70241, I70242, I70243, I70244, I70245, I70248, I70249, I7025, I70261, I70262, I70263, I70268, I70269, I70291, I70292, I70293, I70298, I70299, I70301, I70302, I70303, I70308, I70309, I70311, I70312, I70313, I70318, I70319, I70321, I70322, I70323, I70328, I70329, I70331, I70332, I70333, I70334, I70335, I70338, I70339, I70341, I70342, I70343, I70344, I70345, I70348, I70349, I7035, I70361, I70362, I70363, I70368, I70369, I70391, I70392, I70393, I70398, I70399, I70401, I70402, I70403, I70408, I70409, I70411, I70412, I70413, I70418, I70419, I70421, I70422, I70423, I70428, I70429, I70431, I70432, I70433, I70434, I70435, I70438, I70439, I70441, I70442, I70443, I70444, I70445, I70448, I70449, I7045, I70461, I70462, I70463, I70468, I70469, I70491, I70492, I70493, I70498, I70499, I70501, I70502, I70503, I70508, I70509, I70511, I70512, I70513, I70518, I70519, I70521, I70522, I70523, I70528, I70529, I70531, I70532, I70533, I70534, I70535, I70538, I70539, I70541, I70542, I70543, I70544, I70545, I70548, I70549, I7055, I70561, I70562, I70563, I70568, I70569, I70591, I70592, I70593, I70598, I70599, I70601, I70602, I70603, I70608, I70609, I70611, I70612, I70613, I70618, I70619, I70621, I70622, I70623, I70628, I70629, I70631, I70632, I70633, I70634, I70635, I70638, I70639, I70641, I70642, I70643, I70644, I70645, I70648, I70649, I7065, I70661, I70662, I70663, I70668, I70669, I70691, I70692, I70693, I70698, I70699, I70701, I70702, I70703, I70708, I70709, I70711, I70712, I70713, I70718, I70719, I70721, I70722, I70723, I70728, I70729, I70731, I70732, I70733, I70734, I70735, I70738, I70739, I70741, I70742, I70743, I70744, I70745, I70748, I70749, I7075, I70761, I70762, I70763, I70768, I70769, I70791, I70792, I70793, I70798, I70799, I708, I7090, I7091, I7092, I7100, I7101, I7102, I7103, I711, I712, I713, I714, I715, I716, I718, I719, I720, I721, I722, I723, I724, I725, I726, I728, I729, I7300, I7301, I731, I7381, I7389, I739, I742, I743, I744, I75011, I75012, I75013, I75019, I75021, I75022, I75023, I75029, I7770, I7771, I7772, I7773, I7774, I7775, I7776, I7777, I7779, I790, I791, I798 |
| Transient ischemic attack (TIA) | 435.x | G458, G459, Z8673 |
| Coronary artery disease | 410.xx-414.xx | I200, I201, I208, I209, I2101, I2102, I2109, I2111, I2119, I2121, I2129, I213, I214, I220, I221, I222, I228, I229, I240, I241, I248, I249, I2510, I25110, I25111, I25118, I25119, I252, I253, I2541, I2542, I255, I256, I25700, I25701, I25708, I25709, I25710, I25711, I25718, I25719, I25720, I25721, I25728, I25729, I25730, I25731, I25738, I25739, I25750, I25751, I25758, I25759, I25760, I25761, I25768, I25769, I25790, I25791, I25798, I25799, I25810, I25811, I25812, I2582, I2583, I2584, I2589, I259 |
| Falls | E880-888 | W00-W19 |
| **Clinical Outcome Variables Codes** | | |
| Hemorrhagic Stroke | 430.xx-432.xx. Cases will be excluded if traumatic brain injury (ICD-9: 800-804, 850-854) was present during hospitalization. | I6000, I6001, I6002, I6010, I6011, I6012, I602, I6030, I6031, I6032, I604, I6050, I6051, I6052, I606, I607, I608, I609, I610, I611, I612, I613, I614, I615, I616, I618, I619 |
| Ischemic Stroke | 433.x1, 434.x1, 436 | I6300, I63011, I63012, I63013, I63019, I6302, I63031, I63032, I63033, I63039, I6309, I6310, I63111, I63112, I63113, I63119, I6312, I63131, I63132, I63133, I63139, I6319, I6320, I63211, I63212, I63213, I63219, I6322, I63231, I63232, I63233, I63239, I6329, I6330, I63311, I63312, I63313, I63319, I63321, I63322, I63323, I63329, I63331, I63332, I63333, I63339, I63341, I63342, I63343, I63349, I6339, I6340, I63411, I63412, I63413, I63419, I63421, I63422, I63423, I63429, I63431, I63432, I63433, I63439, I63441, I63442, I63443, I63449, I6349, I6350, I63511, I63512, I63513, I63519, I63521, I63522, I63523, I63529, I63531, I63532, I63533, I63539, I63541, I63542, I63543, I63549, I6359, I636, I638, I639, I6789 |
| Systemic Embolism | 444.x, 445.x | I7401, I7409, I7410, I7411, I7419, I742, I743, I744, I745, I748, I749, I75011, I75012, I75013, I75019, I75021, I75022, I75023, I75029, I7581, I7589 |
| Major Gastrointestinal bleeding event | 456.0, 456.20, 530.82, 531.0x, 531.2x, 531.4x, 531.6x, 532.0x, 532.2x, 532.4x, 532.6x, 533.0x, 533.2x, 533.4x, 533.6x, 534.0x, 534.2x, 534.4x, 534.6x, 535.01, 535.11, 535.21, 535.31, 535.41, 535.51, 535.61, 537.83, 562.02, 562.03, 562.12, 562.13, 568.81, 569.3, 569.85, 578.x; Procedure Code: 44.43 | I8501, I8511, K2211, K226, K250, K252, K254, K256, K260, K262, K264, K266, K270, K272, K274, K276, K280, K282, K284, K286, K2901, K2921, K2931, K2941, K2951, K2961, K2971, K2981, K2991, K31811, K3182, K5521, K5701, K5711, K5713, K5721, K5731, K5733, K5741, K5751, K5753, K5781, K5791, K5793, K625, K6381, K661, K920, K921, K922, K9161, K9162, K91840, K91841 |
| Major Intracranial Hemorrhage (ICH) | 430, 431, 432.0, 432.1, 432.9, , 852.0x, 852.2x, 852.4x, 853.0x | I6000, I6001, I6002, I6010, I6011, I6012, I602, I6030, I6031, I6032, I604, I6050, I6051, I6052, I606, I607, I608, I609, I610, I611, I612, I613, I614, I615, I616, I618, I619, I6200, I6201, I6202, I6203, I621, I629, S06340A, S06341A, S06342A, S06343A, S06344A, S06345A, S06346A, S06347A, S06348A, S06349A, S06350A, S06351A, S06352A, S06353A, S06354A, S06355A, S06356A, S06357A, S06358A, S06359A, S06360A, S06361A, S06362A, S06363A, S06364A, S06365A, S06366A, S06367A, S06368A, S06369A, S064X0A, S064X1A, S064X2A, S064X3A, S064X4A, S064X5A, S064X6A, S064X7A, S064X8A, S064X9A, S065X0A, S065X1A, S065X2A, S065X3A, S065X4A, S065X5A, S065X6A, S065X7A, S065X8A, S065X9A, S066X0A, S066X1A, S066X2A, S066X3A, S066X4A, S066X5A, S066X6A, S066X7A, S066X8A, S066X9A |
| Major Other hemorrhage | 285.1, 360.43, 362.43, 362.81, 363.61, 363.62, 363.72, 364.41, 372.72, 374.81, 376.32, 377.42, 379.23, 423.0x, 596.7x, 599.7x, 602.1x, 620.1, 621.4, 626.2, 626.5, 626.7, 626.8, 626.9, 719.1x, 782.7, 784.7, 784.8, 786.3x, 958.2, 997.02, 998.11; Procedure codes: 99.04 | D62, D7801, D7802, D7821, D7822, E3601, E3602, E89810, E89811, G9731, G9732, G9751, G9752, H05231, H05232, H05233, H05239, H1130, H1131, H1132, H1133, H2100, H2101, H2102, H2103, H31301, H31302, H31303, H31309, H31311, H31312, H31313, H31319, H31411, H31412, H31413, H31419, H3560, H3561, H3562, H3563, H35731, H35732, H35733, H35739, H4310, H4311, H4312, H4313, H44811, H44812, H44813, H44819, H47021, H47022, H47023, H47029, H59111, H59112, H59113, H59119, H59121, H59122, H59123, H59129, H59311, H59312, H59313, H59319, H59321, H59322, H59323, H59329, H9521, H9522, H9541, H9542, I312, I97410, I97411, I97418, I9742, I97610, I97611, I97618, I97620, J9561, J9562, J95830, J95831, L7601, L7602, L7621, L7622, M2500, M25011, M25012, M25019, M25021, M25022, M25029, M25031, M25032, M25039, M25041, M25042, M25049, M25051, M25052, M25059, M25061, M25062, M25069, M25071, M25072, M25073, M25074, M25075, M25076, M2508, M96810, M96811, M96830, M96831, N421, N857, N897, N920, N923, N930, N938, N939, N9961, N9962, N99820, N99821, R040, R041, R042, R0489, R049, R233, R310, R319, R58, T792XXA; Procedure codes: 30230N1, 30230P1, 30233N1, 30233P1, 30240N1, 30240P1, 30243N1, 30243P1, 30250N1, 30250P1, 30253N1, 30253P1, 30260N1, 30260P1, 30263N1, 30263P1 |

**Supplemental Table 2. Predictors of OAC and DOAC Prescription**

|  | **OAC Prescription vs. No OAC Prescription** | | | | **Initiated with DOACs vs. Initiated with Warfarin** | | | |
| --- | --- | --- | --- | --- | --- | --- | --- | --- |
| **Covariates** | **Odds Ratio** | **95% CI** | | **P-value** | **Odds Ratio** | **95% CI** | | **P-value** |
| **Age Category** |  |  |  |  |  |  |  |  |
| **Age** | 0.968 | 0.967 | 0.968 | <.0001 | 0.996 | 0.995 | 0.996 | <.0001 |
| **Sex** | . | . | . | . | . | . | . | . |
| **Male** | 1.036 | 1.028 | 1.044 | <.0001 | 0.980 | 0.968 | 0.991 | 0.0006 |
| **Race/Ethnicity** |  |  |  |  |  |  |  |  |
| **White (Reference)** |  |  |  |  |  |  |  |  |
| **Black** | 0.768 | 0.756 | 0.78 | <.0001 | 0.781 | 0.763 | 0.801 | <.0001 |
| **Other** | 0.934 | 0.918 | 0.95 | <.0001 | 1.248 | 1.214 | 1.282 | <.0001 |
| **US Geographic Region** |  |  |  |  |  |  |  |  |
| **Northeast (Reference)** |  |  |  |  |  |  |  |  |
| **Midwest** | 0.993 | 0.982 | 1.004 | 0.2185 | 0.747 | 0.735 | 0.759 | <.0001 |
| **South** | 0.877 | 0.868 | 0.886 | <.0001 | 1.397 | 1.376 | 1.419 | <.0001 |
| **West** | 0.828 | 0.818 | 0.838 | <.0001 | 0.951 | 0.934 | 0.969 | <.0001 |
| **Other** | 0.800 | 0.732 | 0.874 | <.0001 | 0.747 | 0.655 | 0.853 | <.0001 |
| **Medicaid Dual Eligibility** | 0.711 | 0.705 | 0.717 | <.0001 | 0.785 | 0.774 | 0.796 | <.0001 |
| **Stroke/SE** |  |  |  |  |  |  |  |  |
| **Ischemic Stroke** | 1.939 | 1.895 | 1.984 | <.0001 | 0.773 | 0.751 | 0.796 | <.0001 |
| **SE** | 4.602 | 4.044 | 5.237 | <.0001 | 0.420 | 0.370 | 0.477 | <.0001 |
| **Bleeding** |  |  |  |  |  |  |  |  |
| **Non-major bleeding** | 0.785 | 0.778 | 0.792 | <.0001 | 0.806 | 0.795 | 0.817 | <.0001 |
| **GI bleeding** | 0.434 | 0.418 | 0.45 | <.0001 | 0.726 | 0.681 | 0.773 | <.0001 |
| **ICH** | 0.299 | 0.282 | 0.317 | <.0001 | 0.720 | 0.650 | 0.799 | <.0001 |
| **Other major bleeding** | 0.483 | 0.465 | 0.500 | <.0001 | 0.659 | 0.619 | 0.701 | <.0001 |
| **Baseline Comorbidities** |  |  |  |  |  |  |  |  |
| **Obesity** | 1.329 | 1.316 | 1.343 | <.0001 | 1.064 | 1.049 | 1.079 | <.0001 |
| **Congestive heart failure** | 1.081 | 1.072 | 1.091 | <.0001 | 0.860 | 0.849 | 0.872 | <.0001 |
| **Diabetes** | 0.916 | 0.908 | 0.923 | <.0001 | 0.881 | 0.871 | 0.892 | <.0001 |
| **Hypertension** | 1.095 | 1.081 | 1.109 | <.0001 | 1.055 | 1.034 | 1.075 | <.0001 |
| **COPD** | 0.855 | 0.847 | 0.863 | <.0001 | 0.974 | 0.960 | 0.987 | 0.0001 |
| **Renal disease** | 0.872 | 0.864 | 0.88 | <.0001 | 0.778 | 0.767 | 0.789 | <.0001 |
| **Myocardial Infarction** | 0.966 | 0.954 | 0.978 | <.0001 | 0.891 | 0.875 | 0.907 | <.0001 |
| **Dyspepsia or stomach discomfort** | 0.885 | 0.877 | 0.893 | <.0001 | 0.982 | 0.968 | 0.996 | 0.0144 |
| **Peripheral vascular disease** | 0.886 | 0.877 | 0.896 | <.0001 | 0.821 | 0.808 | 0.834 | <.0001 |
| **Transient ischemic attack** | . | . | . | . | 1.224 | 1.198 | 1.251 | <.0001 |
| **Coronary artery disease** | 0.891 | 0.882 | 0.901 | <.0001 | 1.113 | 1.095 | 1.132 | <.0001 |
| **History of falls** | 0.730 | 0.720 | 0.740 | <.0001 | 0.982 | 0.961 | 1.003 | 0.1003 |
| **Baseline medications** | . | . | . | . | . | . | . | . |
| **ACE/ARB** | 1.276 | 1.265 | 1.286 | <.0001 | 1.077 | 1.064 | 1.09 | <.0001 |
| **Amiodarone** | 0.560 | 0.545 | 0.575 | <.0001 | 1.037 | 0.991 | 1.085 | 0.1197 |
| **Beta blockers** | 1.173 | 1.164 | 1.182 | <.0001 | 0.960 | 0.949 | 0.971 | <.0001 |
| **H2-receptor antagonist** | 0.891 | 0.878 | 0.903 | <.0001 | 0.980 | 0.959 | 1.003 | 0.0856 |
| **Proton pump inhibitor** | 0.934 | 0.926 | 0.942 | <.0001 | 1.014 | 1.001 | 1.027 | 0.03 |
| **Statins** | 1.169 | 1.159 | 1.178 | <.0001 | 1.072 | 1.060 | 1.085 | <.0001 |
| **Anti-platelets** | 0.876 | 0.866 | 0.886 | <.0001 | 1.054 | 1.036 | 1.072 | <.0001 |
| **NSAIDS** | 1.109 | 1.099 | 1.119 | <.0001 | 1.109 | 1.095 | 1.124 | <.0001 |
| **Healthcare utilizations** | . | . | . | . | . | . | . | . |
| **Inpatient Admission Visit** | 0.945 | 0.937 | 0.953 | <.0001 | 0.815 | 0.805 | 0.826 | <.0001 |
| **ER Visit** | 0.950 | 0.942 | 0.957 | <.0001 | 0.982 | 0.97 | 0.995 | 0.0049 |
| **# of Office Visit** | 1.002 | 1.002 | 1.002 | <.0001 | 1.006 | 1.005 | 1.006 | <.0001 |

**Supplemental Table 3. Interaction of Age on the Associations of History of Ischemic Stroke, Systemic Embolism, and Falls on OAC Therapy Prescription**

| **Category** | **Odds Ratio (95% CI)** | **p-value interaction (Type-3 test)** |
| --- | --- | --- |
| Ischemic Stroke among age: 65-74 | 1.74 (1.67-1.82) | <.0001 |
| Ischemic Stroke among age: 75-84 | 1.86 (1.79-1.93) |  |
| Ischemic Stroke among age: ≥85 | 2.22 (2.14-2.31) |  |
| Systemic embolism among age: 65-74 | 3.13 (2.53-3.88) | <.0001 |
| Systemic embolism among age: 75-84 | 4.24 (3.39-5.31) |  |
| Systemic embolism among age: ≥85 | 7.61 (6.03-9.60) |  |
| Falls among age: 65-74 | 0.80 (0.78-0.82) | <.0001 |
| Falls among age: 75-84 | 0.69 (0.68-0.71) |  |
| Falls among age: ≥85 | 0.71 (0.70-0.73) |  |

**Supplemental Table 4. Interaction of Age on the Associations of History of Ischemic Stroke, Systemic Embolism, ICH Bleed, and Renal Disease on DOAC versus Warfarin Therapy Prescription**

| **Category** | **Odds Ratio (95% CI)** | **P-value Interaction**  **Type-3 test** |
| --- | --- | --- |
| Ischemic Stroke among age: 65-74 | 0.74 (0.70-0.78) | .0676 |
| Ischemic Stroke among age: 75-84 | 0.78 (0.75-0.82) |  |
| Ischemic Stroke among age: ≥85 | 0.80 (0.76-0.85) |  |
| Systemic Embolism among age: 65-74 | 0.41 (0.32-0.51) | .9324 |
| Systemic Embolism among age: 75-84 | 0.43 (0.35-0.53) |  |
| Systemic Embolism among age: ≥85 | 0.42 (0.34-0.53) |  |
| ICH Bleed among age: 65-74 | 0.60 (0.50-0.73) | .0022 |
| ICH Bleed among age: 75-84 | 0.69 (0.60-0.81) |  |
| ICH Bleed among age: ≥85 | 1.00 (0.80-1.25) |  |
| Renal disease among age: 65-74 | 0.67 (0.65-0.68) | <.0001 |
| Renal disease among age: 75-84 | 0.80 (0.78-0.81) |  |
| Renal disease among age: ≥85 | 0.91 (0.89-0.94) |  |
